# “Right on all Occasions?” – On the Feasibility of Laterality Research Using a Smartphone Dichotic Listening Application

**DOI:** 10.3389/fpsyg.2013.00042

**Published:** 2013-02-07

**Authors:** Josef J. Bless, René Westerhausen, Joanne Arciuli, Kristiina Kompus, Magne Gudmundsen, Kenneth Hugdahl

**Affiliations:** ^1^Department of Biological and Medical Psychology, University of BergenBergen, Norway; ^2^Division of Psychiatry, Haukeland University HospitalBergen, Norway; ^3^Faculty of Health Sciences, University of SydneySydney, NSW, Australia; ^4^Department of Radiology, Haukeland University HospitalBergen, Norway

**Keywords:** laterality, dichotic listening, language lateralization, smartphone, mobile device, software application

## Abstract

Most psychological experimentation takes place in laboratories aiming to maximize experimental control; however, this creates artificial environments that are not representative of real-life situations. Since cognitive processes usually take place in noisy environments, they should also be tested in these contexts. The recent advent of smartphone technology provides an ideal medium for such testing. In order to examine the feasibility of mobile devices (MD) in psychological research in general, and laterality research in particular, we developed a MD version of the widely used speech laterality test, the consonant-vowel dichotic listening (DL) paradigm, for use with iPhones/iPods. First, we evaluated the retest reliability and concurrent validity of the DL paradigm in its MD version in two samples tested in controlled, laboratory settings (Experiment 1). Second, we explored its ecological validity by collecting data from the general population by means of a free release of the MD version (*iDichotic*) to the iTunes App Store (Experiment 2). The results of Experiment 1 indicated high reliability (*r*_ICC_ = 0.78) and validity (*r*_ICC_ = 0.76–0.82) of the MD version, which consistently showed the expected right ear advantage (REA). When tested in real-life settings (Experiment 2), participants (*N* = 167) also showed a significant REA. Importantly, the size of the REA was not dependent on whether the participants chose to listen to the syllables in their native language or not. Together, these results establish the current MD version as a valid and reliable method for administering the DL paradigm both in experimentally controlled as well as uncontrolled settings. Furthermore, the present findings support the feasibility of using smartphones in conducting large-scale field experiments.

## Introduction

Traditionally, the laboratory functions as center stage for psychological experiments in general, and laterality research in particular. Although this has obvious advantages, it is often too resource demanding to reach a larger audience and obtain a broad sample. In experimental psychological research the control of confounding variables is weighed against the degree of ecological validity; usually aiming to maximize control at the expense of ecological validity (Brunswik, 1947). However, the advent of handheld mobile devices (MDs; e.g., smartphones) with processing power comparable to stationary systems has opened the door to transferring experiments from the laboratory to real-life settings while maintaining control over stimulus presentation. In real-life, cognitive processes are executed in noisy environments. Thus, the natural environment is the authentic arena where psychological theories can be proven to transcend laboratory walls and stand the test of real-life situations. This approach is not entirely new; however, until recently, it has been promoted mainly within a clinical context where it is referred to as ambulatory assessment involving the acquisition of psychophysiological data and self-reports in natural settings (e.g., Fahrenberg, 1996). While the popularity of internet-based psychological testing has grown rapidly over the last decade (see, Barak and Buchanan, 2004), the use of MDs for data collection is still in its infancy. One clear advantage of using MDs over internet-based testing that relies mostly on stationary computers is the possibility to access participants over the whole day, anywhere that they happen to be at that particular time, allowing for unique opportunities for experimental intervention. Some recent studies have harnessed this advantage by acquiring participants’ self-reports on their current mood (Courvoisier et al., 2010) as well as their cognitive performance at controlled time points during the day (Tiplady et al., 2009; Kennedy et al., 2011). While these studies include a fixed sample with a mainly clinical focus, there are also those that use open “recruitment” of participants through a software application that can be downloaded and consequently reach a larger audience (crowd sourcing) than what is normally achieved with common sampling methods (e.g., Killingsworth and Gilbert, 2010; Dufau et al., 2011). A review of various types of behavioral data collection using smartphone technology and their limitations is presented by Miller (2012).

The objective of the present experiments was to examine the feasibility of paradigms implemented via MDs for the purposes of laterality research. For this purpose, we chose a classical speech laterality test, namely, dichotic listening (DL; Bryden, 1988; Hugdahl, 2003, 2011); a test which has been used in laboratories around the world for decades (see, Hugdahl, 2011). The history of the DL paradigm in laterality research goes back half a century to research conducted by Kimura (1961, 2011), who found that when simultaneously presented with two verbal stimuli, one to the left ear (LE) and the other to the right ear (RE), participants exhibit the tendency to report the RE stimulus more often than the LE stimulus (the so-called RE advantage, REA). This finding is commonly interpreted as an indicator of left hemisphere processing of language (e.g., Kimura, 1967; Pollmann, 2010). Support for this interpretation of the REA comes from studies using functional magnetic resonance imaging (e.g., Jäncke et al., 2002; van den Noort et al., 2008), positron emission tomography (e.g., O’Leary et al., 1996; Hugdahl et al., 1999), electroencephalography (e.g., Brancucci et al., 2004), magnetoencephalography (e.g., Alho et al., 2012), Wada-test (e.g., Hugdahl et al., 1997), as well as from studies on split brain patients and patients with callosal lesion (e.g., Milner et al., 1968; Springer and Gazzaniga, 1975; for a review see Westerhausen and Hugdahl, 2008). There are a number of variants of the DL test mainly differing in the stimulus material used. In the present study, we used the consonant-vowel (CV) paradigm (Shankweiler and Studdert-Kennedy, 1967; Hugdahl and Andersson, 1986), which according to a meta-analysis by Voyer (1998) produces the most reliable laterality effects, with reliability ranging from 0.61 (Bryden, 1975; split-half reliability, Spearman *r*) to 0.91 (Wexler et al., 1981; test-retest, Pearson *r*).

For the present project, we developed a MD version of the DL test (*iDichotic*) for the iPhone/iPod touch and tested it in two steps. First, we used it in a controlled laboratory setting where we evaluated the validity and reliability of the DL paradigm in its MD version (Experiment 1). Second, we investigated whether the MD version produces robust results when applied to the general population as part of a “crowd sourcing” field experiment (Experiment 2), by making the paradigm publicly available on Apple’s digital application distribution platform (App Store).

## Experiment 1

In the first experiment, reliability of the MD version of the DL paradigm was assessed in a Norwegian sample as well as an Australian sample, to test the intercultural transfer of results. For this purpose, we adopted a test-retest design according to Cohen et al. (1996), in which participants were tested twice with the same version of the paradigm and performing the same task, and then calculated the correlation of laterality indices from each time point. In addition, concurrent validity of the MD version was tested by using the results of the standard personal computer (PC) version as “criterion.” The results of the PC version were used as criterion since it represents the current standard procedure for measuring speech laterality as conducted in our laboratories and most others (Hugdahl, 2003).

### Materials and Methods

#### Participants

The Norwegian sample included 33 healthy, subjects with a mean age of 31.7 years (SD = 9.8) including 22 female and 11 male participants. The Australian sample included 43 healthy, female subjects with a mean age of 21.6 years (SD = 2.7). The exclusion criteria were as follows: left-handedness (self-report), more than three homonym errors (see below), less than six overall correct reports, and more than 20% hearing asymmetry at either time point (inferred from hearing test results administered as part of the application). Participants gave written informed consent.

#### Material and procedure

The stimulus material was based on the standard Bergen DL paradigm (Hugdahl, 2003), using the six CV syllables/ba/, /da/, /ga/, /ta/, /ka/, and /pa/ as stimulus material. The stimuli were pairwise, dichotically presented CV syllables via headphones/earphones, and in all possible pairwise combinations yielding a total of 36 pairs, also including six homonym pairs with the same syllable presented to the LE and RE. The syllables used for the Norwegian sample were spoken by a native, male Norwegian speaker with constant intonation and intensity, and had a mean duration between 400–500 ms. Likewise, the Australian sample was correspondingly tested with syllables spoken by a native, male English speaker, and had a mean duration between 480–550 ms. The syllables in each pair were temporally aligned to each other for simultaneous onset of their initial stop-consonants. The MD version included a hearing test to control for hearing asymmetries, which can bias the results toward the right or LE. In this test the loudness of a 1000 Hz tone had to be regulated using a horizontal volume scroll bar to indicate when tone is just inaudible (separate for LE and RE).

In the Norwegian sample each participant completed the test four times, twice as the standard PC version, and twice using the MD version (see below). The order of the four test runs was inter-individually balanced using an ABBA design. Participants in the Australian sample undertook two consecutively presented test runs only using the MD version of the paradigm.

For both samples, a test run consisted of the presentation of a full set of 36 stimulus pairs, which were pseudo-randomly presented with a 4000 ms inter-stimulus interval. Within the interval between stimulus presentations participants were asked to respond manually, either by key press for the PC implementation or by using the touch screen of the MD. There were six labeled buttons on the keyboard and six buttons on the touch screen, respectively, one for each syllable used in the test. Regardless of mode of implementation only one answer was possible per trial. The instructions followed free-report instruction (non-forced condition, cf. Hugdahl, 2003); that is, participants were instructed to listen to the syllables and report after each trial which syllable they heard best. An answer was considered to be “correct” when the response matched either right or the LE stimulus in that particular trial; it was counted as “error” when the chosen syllable had not been presented or when no response was given. The subjects did not get feedback about their performance until the end of the experiment.

Stimulus administration was delivered via Sennheiser headphones for the PC version and via the standard Apple earphones for the MD version. In view of the potential for differences in the quality of the output, especially with regard to the possibility of asymmetric presentation of the stimuli, we recorded a white noise spectrogram from the two types of headphones. The right-left mean differences within the frequencies relevant for speech (250 Hz–2 kHz) were −0.12 dB for the Sennheiser headphones and 0.32 dB for the Apple earphones. In light of previous research, showing that only inter-aural differences above 6 dB affect the magnitude of the ear advantage (Hugdahl et al., 2008), we considered the present differences of well below 1 dB to be negligible.

For each test run, the number of correct responses of LE and RE stimuli was recorded and used to determine a laterality index (LI) calculated according to the following formula: LI = [(RE − LE)/(RE + LE)] × 100. Thus, the LI expresses the percentage difference between the correct LE and RE reports with positive values indicating a right, and negative values a LE advantage.

#### Instruments

The PC version of the CV-DL paradigm was programmed and run in E-prime (Version 2; Psychology Software Tools, http://www.pstnet.com/). The MD version was developed in Xcode 3.2.5 using the iOS software development kit (Apple Inc., Cupertino, CA) and administered on iPhone or iPod touch units running as a prototype version of the final *iDichotic* application (see Experiment 2).

#### Statistical analysis

Intraclass correlation analyses [ICC(3,1), see Shrout and Fleiss, 1979] were conducted to determine reliability and validity of the MD version. For data from both samples, reliability was determined as retest reliability and obtained by correlating the LI of the two test runs using the MD version. Additionally, for the Norwegian sample, reliability was calculated for the results of the PC version. Validity of the MD version was assessed within the Norwegian sample data by calculating the intraclass correlation between the results of the two test runs with the MD version and the results of the standard PC version. Here, the mean LI of the two test runs via the PC version was used as criterion.

Additional analyses were conducted in order to test for mean differences between the two DL versions and the effect of test repetition on the LI (dependent variable). In the Norwegian sample, a 3-way analysis of variance (ANOVA) with within-subject factors *Version* and *Timepoint*, as well as between-subject factor *Sex*. Comparably, for the Australian sample, a *t*-test was calculated to compare the mean LI across the two test runs. The above analyses were supplemented with one-sample *t*-tests against zero to test for significant LI, i.e., REA, and an independent-samples *t*-test comparing the total mean LI of the Norwegian sample with the total mean LI of the Australian sample. In order to further investigate the differences between the samples, we conducted two *post hoc* analyses. First, to examine possible sex effects, only the females of both groups were compared. Second, to address possible effects of the presentation device, only the results collected with the MD version were compared.

For all analyses, level of significance was set to α = 0.05 and effect sizes were provided as measures of explained variance (η^2^), or as standardized mean difference (Cohen’s *d*). Statistical analyses were performed in PASW 18.0 (IBM SPSS, New York, USA).

### Results

**Figure 1:**
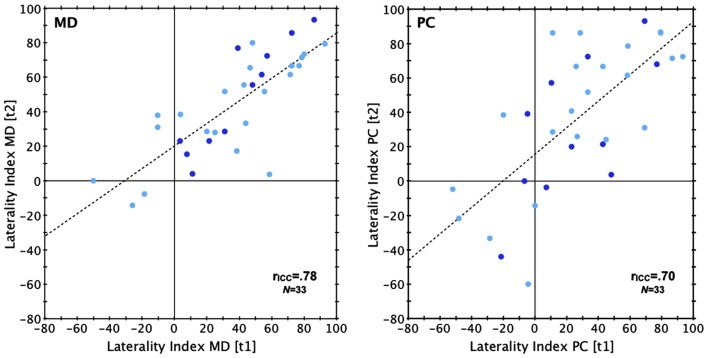
**Reliability (Norwegian sample)**. Scatterplot depicting intraclass correlations between results at test run 1 (t1) and test run 2 (t2; left: MD version; right: PC version). Laterality index, percentage difference between correct LE and RE reports. r_ICC_, intraclass correlation coefficient. Dot color indicates sex: light blue, females; dark blue, males.

**Figure 2:**
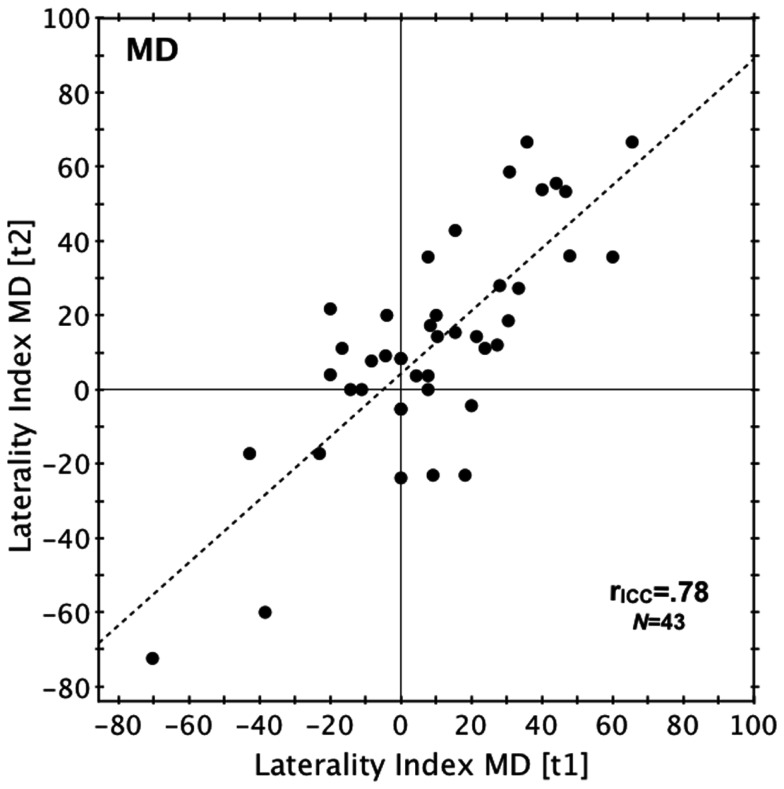
**Reliability (Australian sample)**. Scatterplot relating the LI of the first and second test run in the Australian sample. Laterality index, percentage difference between correct LE and RE reports. r_ICC_, intraclass correlation coefficient.

**Figure 3:**
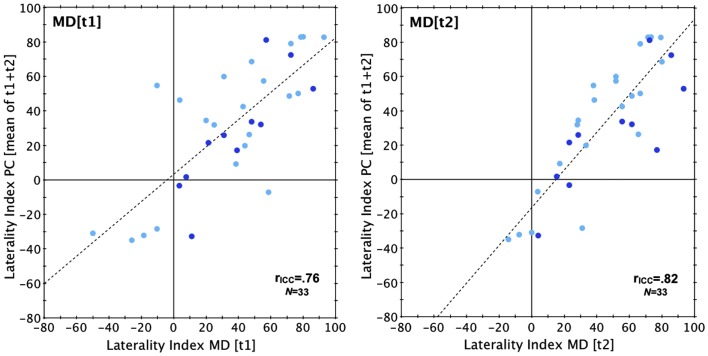
**Validity**. Scatterplot showing the results yielded with MD version at test run 1 (left) and 2 (right) when related to the aggregated results obtained with the PC version. Laterality index, percentage difference between correct LE and RE reports. r_ICC_, intraclass correlation coefficient. Dot color indicates sex: light blue, females; dark blue, males.

The retest reliability was identical in both the Norwegian and the Australian sample (both *r*_ICC_ = 0.78) and slightly higher than the reliability of the PC version (*r*_ICC_ = 0.70; Norwegian sample only; see also Figures 1 and 2). Validity, tested in the Norwegian sample by correlating the results of MD and PC version (see Figure 3) was slightly higher for test run 2 (*r*_ICC_ = 0.82) than for test run 1 (*r*_ICC_ = 0.76).

The ANOVA conducted for the Norwegian sample revealed main effects of *Version* [*F*(1,31) = 8.64, *p* = 0.01, η^2^ = 0.023, MD > PC] and *Timepoint* [*F*(1,31) = 4.40, *p* = 0.04, η^2^ = 0.014, test run 2 > test run 1]. Neither the interaction of the within-subjects factors [*F*(1,31) = 0.004, *p* = 0.81, η^2^ < 0.001], nor the main effect of the between-subject factor of Sex [*F*(1,31) = 0.001, *p* = 0.98, η^2^ < 0.001] were significant. In the Australian sample there was no significant difference between the two test runs [*t*(42) = −1.10, *p* = 0.28, *d* = −0.11].

A REA was found for both versions of the DL paradigm and in both samples. In the Norwegian sample, the MD version produced a LI of 36.5% ± 35.3 (test run 1) and 44.2% ± 29.3 (test run 2), while the PC version produced a LI of 27.2% ± 38.5 (test run 1) and 36.3% ± 41.9 (test run 2). Each of these LIs was significantly larger than zero [all *t*(32) > 4.06, all *p* < 0.001, *d* = 0.71–1.51]. As for the Australian sample, the LI was 9.2% ± 27.2 (test run 1) and 12.3% ± 29.4 (test run 2), both significantly larger than zero [test 1: *t*(42) = 2.21, *p* = 0.03, *d* = −0.34; test 2: *t*(42) = 2.75, *p* = 0.01, *d* = 0.42]. For an overview of the correct ear scores and laterality indices for both samples see Table A1 in Appendix. A comparison of the mean LI across all test runs and versions of the Norwegian sample (LI = 36.0% ± 32.5) against the mean LI across both test runs of the Australian sample (LI = 10.8 ± 26.8) revealed that the Norwegian sample had a significantly stronger REA [*t*(74) = 3.7, *p* < 0.01, *d* = 0.85]. Comparing only the females of both samples still showed a significantly larger LI in the Norwegian sample [Norwegian sample: 36.1% ± 34.5; Australian sample: 10.8 ± 26.8; *t*(63) = 3.3, *p* < 0.01, *d* = 0.82]. Also when only MD results were compared, the Norwegian sample had a significantly larger LI [Norwegian sample: 40.3% ± 30.6; Australian sample: 10.8 ± 26.8; *t*(74) = 4.5, *p* < 0.001, *d* = 1.03].

### Discussion

The results from the Norwegian and Australian samples indicate that the MD version of the DL paradigm produces highly reliable results, with intraclass correlation coefficients slightly higher than that obtained via the PC version in the Norwegian sample. With an intraclass correlation of 0.78 the reliability of the MD version is well within the range usually found in studies using CV DL paradigms (i.e., between 0.61 and 0.91, cf. Voyer, 1998). Hugdahl and Hammar (1997), using the same DL paradigm on a Walkman, showed a medium-strong correlation coefficient of 0.61. The authors used a test-retest interval of 2 weeks compared to the present consecutive administration, which may explain the higher correlation in the present study. We also assessed criterion validity in the Norwegian sample and it appears to be high, as indicated by strong correlations between the results of both MD-based test runs along with the results obtained with the standard PC version.

Beyond demonstrating high reliability and validity, the findings revealed some results that deserve further discussion. First, as indicated by a significant main effect in the Norwegian sample, the second test run produced a stronger REA than the first, irrespective of whether MD or PC version was applied. This effect might be due to practice, habituation effects, or a general familiarization with stimulus material and testing procedure. For example, practice effects have been shown to increase performance and reverse laterality in a mental rotation task (Voyer et al., 1995). Nevertheless, the *Timepoint* effect was small (2.3% explained variance) and was not replicated in the larger Australian sample.

A second interesting observation in the Norwegian sample was that the MD produced a stronger REA than the PC version. However, this effect was also small, accounting for only 2% of the variance in the dependent variable. Assuming that the MD and PC version did not produce a systematic effect on laterality in terms of output level (see spectrogram test in Materials and Methods section), one possible reason for the version effect might be found by considering the responses that were required. While the MD version required participants to hold the device in the right hand and respond with the right thumb, the PC version used response keys distributed on a keyboard to be used with fingers of the right hand. This might result in differential demands for the visual-motor coordination, differentially favoring left or right hemispheric processing, and thus indirectly affecting the laterality as measured with the DL paradigm. However, without further evidence any such interpretation remains speculative, and as pointed out above, the effect was rather small, hence not substantially affecting the reliability measures which, calculated as ICC(3,1), also incorporate mean differences in the reliability calculations (cf. Shrout and Fleiss, 1979).

Finally, the MD version in the female-only, Australian sample produced a smaller REA than both versions in the Norwegian sample, suggesting that factors such as native language background and sex of the subjects may contribute to the magnitude of the REA. Indeed, a comparison of the mean LI obtained with similar DL studies conducted in several countries with different languages, indicates that the REA might be smaller in English speakers [LI of about 14% in Hirnstein (2011)] than in Norwegian (about 26%, Rimol et al., 2006) or German speakers (about 30%; Westerhausen et al., 2006). With regard to sex, the REA is frequently found to be more pronounced in male as compared to female subjects (e.g., Lake and Bryden, 1976; Zatorre, 1979; Cowell and Hugdahl, 2000; for a review see Voyer, 2011). Thus, in view of differences in both the sex distribution and language background across the two samples, a stronger LI in the Norwegian sample would be predicted. However, the present analyses also revealed a significant difference between the Australian and Norwegian sample when only results of the female participants were compared, indicating that sex alone is insufficient in explaining the difference between the two samples. Based on this observation, Experiment 2 was conducted to further examine the possible effects of language background and sex on the MD results.

## Experiment 2

In the second experiment, data was collected from volunteer users around the world who submitted their test results to a database via the mobile DL application (*iDichotic*). The main aim was to explore if smartphones can produce comparable results in the field as well as in the laboratory and thus be suitable as platforms for large-scale population studies. In particular, we investigated the question of sound language, first as to whether the choice of sound in relation to language background (congruent: Norwegian and English native speakers who also chose their native sound vs. incongruent: participants with various language backgrounds who had to select a non-native sound) influences the results, with implications for the number of native sounds one should provide; and second, as a follow-up to the results of the first experiment, as to whether English and Norwegian syllables selected by native English speakers and native Norwegian speakers, respectively, produce significantly different LIs in this larger sample.

### Materials and Methods

#### Participants

The *iDichotic* application was promoted via various media channels (e.g., university news, websites, TV) and word-of-mouth resulting in 508 downloads over the course of 5 months (between release of the application on 11th December 2011 and 11th May 2012). In total, 263 results were submitted (i.e., 52% of those who downloaded the app chose to submit their results). After applying the exclusion criteria, 167 participants were included in the study (see Table 1 for details). This constitutes the main sample and is the basis for exploring whether the choice of native sound vs. non-native sound has an effect on the results. In addition, a sub-sample of *N* = 107 participants, including only self-reported native speakers of either Norwegian or English who also selected their native language as sound language (see Table 1), served as the basis for investigating whether the differences in LIs found between Norwegian and English samples of Experiment 1 also emerge in this larger field data.

**Table 1:** **Sample characteristics Experiment 2**.

		*N*	Sex		Age (mean ± SD)
			Male	Female	
Stimulus-Language Congruency^a^ (analysis 1)	YES	108	69	39	34.1 (±12.6)
	NO	59	38	21	30.5 (±12.2)
	Σ	167	107	60	32.8 (±12.6)
Sound Language^b^ (analysis 2)	NOR	78	55	23	32.5 (±11.2)
	ENG	30	14	16	38.3 (±15.1)
	Σ	108	69	39	34.1 (±12.6)

**N*, number of subjects; SD, standard deviation*.

*^a^*Yes*, subject selected native sound; *No*, subject did not select native sound; Σ, sum*.

*^b^*NOR*, Norwegian native speaker that selected Norwegian as sound language; *ENG*, English native speaker that selected English as sound language*.

The following exclusion criteria were applied to the dataset: more than three errors in the identification of homonyms, less than six correct reports, more than 20% hearing asymmetry (deduced from hearing test results implemented in the application, see below), and other-than-first submissions from the same participant, left-handedness, or ambidexterity (self-reported under settings).

#### Material

The *iDichotic* application (v. 1.1.0) was the same as the pre-release version used in Experiment 1 with some minor graphical and functional changes concerning the presentation and submission of results.

After downloading and installing the application on their MD, the participants were first directed to the settings page of the application, where they had to select a sound language (Norwegian or English), fill out information about themselves (age, sex, handedness, and native language), as well as perform a hearing test. In this test the loudness of a 1000 Hz tone had to be regulated using a horizontal volume scroll bar to indicate when tone is just inaudible (separate for LE and RE). When these settings were completed, participants could start with the DL task (termed “Listen” test in the application). A pop-up notification reminded the user to wear the earphones in correct ears and check the main volume. Instructions were presented on the screen prompting the user to listen to a series of syllables and report after each trial (by using buttons on the touch screen) the syllable he/she heard best. At completion of the test, which takes approximately 3 min, the results were displayed and the option to submit the data package (see below) to our database was presented.

#### Data collection

The voluntarily submitted user data package was collected via secure file transfer protocol and stored on the servers at University of Bergen. The data packages were anonymous and included the results, user settings, and submission date, as well as an application-ID (date of application download + random number), which allowed for the exclusion of double submissions. Informed consent was obtained before submission of results by means of a pop-up text window which prompted the user to submit or close.

#### Statistical analysis

In the main sample, a two-way ANOVA was conducted with LI as the dependent variable (see Experiment 1) and the between-subjects factors of *Sex* and *Stimulus-Language Congruenc*y. A second two-way ANOVA was conducted in a sub-sample (for sample characteristics, see Table 1) with LI as the dependent variable (see Experiment 1) and the between-subject factors *Sex* and *Sound Language*. The level of significance was set to α = 0.05 and effect sizes were calculated as η^2^ and *d*, respectively. The analysis was performed in PASW 18.0 (IBM SPSS, New York, USA). Power analysis was performed using GPower 3.0 (Faul et al., 2007).

### Results

**Figure 4:**
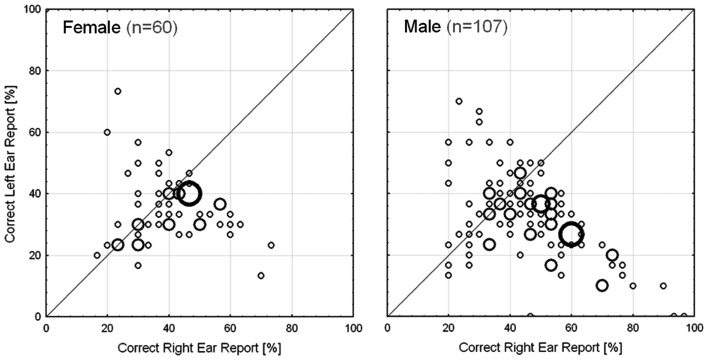
**Distribution of the field data separated by sex; collected via *iDichotic***. Size of bubble reflects the number of subjects with the same ear scores (displayed as percentage correct reports of right and left ear stimulus in one test run). Left: female subjects; right: male subjects.

The first ANOVA revealed a significant main effect of *Sex* [*F*(1,163) = 4.76, *p* = 0.031, η^2^ = 0.028] with males having a stronger LI than females (males: 17.6% ± 30.8; females 4.7% ± 25.2). Neither the main effect of *Stimulus-Language Congruenc*y [*F*(1,163) = 0.50, *p* = 0.480, η^2^ = 0.003] nor the interaction was significant [*F*(1,163) = 2.64, *p* = 0.106, η^2^ = 0.015]. The statistical power of the test for the non-significant main and interaction effect of stimulus-language congruency was with 0.83 sufficiently high to exclude population effect explaining more than 5% of the variance. Finally, a significant intercept [*F*(1,163) = 23.02, *p* < 0.001] indicated a significant REA in the sample (mean LI = 13.0% ± 29.5; *d* = 0.44). Subjects that selected their native sound language displayed a mean LI of 12.5% ± 32.5 compared to 13.8% ± 23.2 of those who did not select their native sound language. Fifty-three out of 59 (89.8%) non-English/non-Norwegian native speakers selected English as the sound language. The distribution of correct RE and LE reports are shown in a scatterplot in Figure 4.

In line with the results of the first ANOVA, the second ANOVA revealed a significant main effect of *Sex* [*F*(1,104) = 7.03, *p* = 0.009, η^2^ = 0.063] with males showing a stronger LI than females. Neither the main effect of *Sound* *Language* [*F*(1,104) = 1.20, *p* = 0.277, η^2^ = 0.011] nor the interaction was significant [*F*(1,104) = 0.31, *p* = 0.581, η^2^ = 0.003]. The statistical power of the test for the main effect of sound language was with 0.80 sufficiently high to exclude population effect explaining more than 7% of the variance. Finally, a significant intercept [*F*(1,104) = 6.53, *p* = 0.012] indicated a significant REA in the sub-sample (mean LI = 12.5% ± 32.5; *d* = 0.38).

### Discussion

Utilizing a MD DL test we collected data in a large international field experiment and were able to replicate the REA usually found with this paradigm (e.g., Studdert-Kennedy and Shankweiler, 1970; Hugdahl and Andersson, 1984), supporting the usability of MDs as “mobile laboratories.” Furthermore, we also observed a significant effect of sex, with males displaying a larger REA than females. This finding is in line with a frequently observed stronger behavioral laterality in males (e.g., McGlone, 1980). However, recent meta-analytic evidence (e.g., Voyer, 2011; see also Hiscock et al., 1994) as well as studies utilizing larger study samples (Hirnstein et al., in press), indicate that the sex effect found with DL is rather small, explaining about 1% of the variance in laterality. Against this background, the larger sex effect found in Experiment 2 (2.8% explained variance in the complete sample) is likely due to a sampling bias.

Since large-scale field experiments like this include participants from many backgrounds and not all native sounds can be provided, the question was raised as to whether selecting a non-native sound would have an effect on the ear advantage. This is an important issue because on it depends whether non-natives to a selected sound have to be excluded from the analysis. The results from the first ANOVA showed that also non-native speakers might be included in the analysis, suggesting that lack of non-native materials is not necessarily a hindrance in world-wide data collections.

Based on the findings from Experiment 2, it appears that language background cannot explain the differences observed in Experiment 1, although the same trend toward larger LI in the Norwegian sample compared to the English sample is seen in the present experiment as well as in previous studies (see Discussion of Experiment 1).

## General Discussion

The objective of the experiments reported here was to examine the feasibility of MD applications in laterality research. Having established the validity and reliability of the MD version under controlled conditions in the laboratory (Experiment 1), we examined how the MD application performed in uncontrolled conditions in the field (Experiment 2), where circumstances surrounding self-administration of the test are unknown (e.g., environmental noise, location, headphone quality, subject’s state of mind etc.). For example, as seen in an earlier study, background noise can significantly reduce the REA (Dos Santos Sequeira et al., 2010) and thus might also have an effect on the present field data. Despite these issues, the results displayed a significant REA suggesting that laboratory experiments can be replicated in real-life settings via MDs. In addition, the REA appears to be “robust” enough to resist “noise” factors. Thus, the present MD application appears to be a valid and reliable alternative to the traditional method of administering DL on a PC, independent of the experimental setting.

The field experiment results further imply that heterogeneity of a sample should not always be avoided, especially when the aim is to test universal theories of the brain. Other examples for this kind of sampling approach are a study on lexical decisions by Dufau et al. (2011) and another study on mind wandering and mood by Killingsworth and Gilbert (2010), both employing smartphone technology to collect data from users world-wide. Analogous to our experiment, the authors used Apple’s App Store for distribution of the application.

The results from both experiments show that although a significant REA was found in all samples, there are also variations between them. The Norwegian sample in Experiment 1 appears to stand out as particularly RE-biased whereas all other samples, including the Norwegian sub-sample in Experiment 2, displayed smaller REAs. This cannot be solely explained by the different sex distributions of the samples, although sex appears to have an effect on speech laterality, as seen in previous studies (e.g., Hirnstein et al., in press; Voyer, 2011; see also Discussion under Experiment 2) as well as in the present Experiment 2. Also language background is not a sufficient factor in explaining the laterality differences observed Experiment 1, since there was no significant effect of sound language in Experiment 2, although previous studies have suggested such a link (see Discussion above). In summary, the variations we see may be due to a combination of factors, that is sex (to a lesser degree) or sound language.

### Lessons for future smartphone field experiments

Given that environment/background noise can have a significant influence on test results (Dos Santos Sequeira et al., 2010), one should consider collecting data on the circumstances surrounding the testing. For example, the participants could be asked to provide information about their location, or the microphone built into the MD could be used to determine the background noise level. Also data on the hardware (device, headphones) and software version used for the test may be useful information, especially if the test runs on various platforms. One should be aware of systematic errors introduced by different hardware/software, e.g., bias toward one output channel (ear); however, currently, *iDichotic* is limited to Apple’s MDs that run iOS software version 5 or later, and we are not aware of any systematic differences between the versions that might have affected our results.

## Conclusion

Taken together, as here demonstrated regarding the REA in DL, current smartphone technology allows for a validation of laterality phenomena and cognitive constructs in the field. Validation of our mobile application in patients who cannot visit research facilities, for example, hospitalized patients undergoing neuropsychological assessment, is a logical next step. Also, studies designed to investigate longitudinal changes, such as infradian effects of sex hormones like estradiol (e.g., Cowell et al., 2011; Hjelmervik et al., 2012) on laterality, or symptoms-related cognitive fluctuations (e.g., Green et al., 1994; Escandon et al., 2010), as well as molecular genetic studies with the need to recruit large cohorts (e.g., Ocklenburg et al., 2011) could benefit from data collection using MDs.

## Conflict of Interest Statement

The authors declare that the research was conducted in the absence of any commercial or financial relationships that could be construed as a potential conflict of interest.

## Appendix

**Table A1:** **Correct report (mean ± standard deviation) for each sample, test version, and timepoint**.

		*t*1			*t*2		
		LE	RE	LI	LE	RE	LI
NOR	PC	32.5 (±16.9)	58.0 (±19.4)	27.2 (±38.3)	27.9 (±17.7)	62.0 (±21.8)	36.3 (±41.9)
	MD	28.8 (±16.3)	62.3 (±17.7)	36.5 (±35.3)	25.5 (±12.3)	67.1 (±15.7)	44.2 (±29.3)
AUS	MD	36.2 (±12.3)	44.0 (±14.0)	9.2 (±27.2)	37.6 (±14.0)	48.8 (±15.6)	12.3 (±29.4)

*t1/t2, first and second testing, respectively. LE, left ear; RE, right ear; LI, laterality index; NOR, Norwegian sample; AUS, Australian sample*.
